# Team Building Through Team Video Games: Randomized Controlled Trial

**DOI:** 10.2196/28896

**Published:** 2021-12-14

**Authors:** Mark J Keith, Douglas L Dean, James Gaskin, Greg Anderson

**Affiliations:** 1 Information Systems Department Marriott School of Business Brigham Young University Provo, UT United States

**Keywords:** team video gaming, team building, flow, team cohesion, video games, gamification, team, teamwork, cohesion, theory, framework, performance

## Abstract

**Background:**

Organizations of all types require the use of teams. Poor team member engagement costs billions of US dollars annually.

**Objective:**

This study aimed to explain how team building can be accomplished with team video gaming based on a team cohesion model enhanced by team flow theory.

**Methods:**

In this controlled experiment, teams were randomly assigned to a team video gaming treatment or a control treatment. Team productivity was measured during both pretreatment and posttreatment team tasks. After the pretest, teams who were involved in the team video gaming treatment competed against other teams by playing the Halo or Rock Band video game for 45 minutes. After the pretest, teams in the control treatment worked alone for 45 minutes. Then, all teams completed the posttest team activity. This same experimental protocol was conducted on 2 different team tasks.

**Results:**

For both tasks, teams in the team video gaming treatment increased their productivity significantly more (*F*_1_=8.760, *P*=.004) on the posttest task than teams in the control treatment. Our flow-based theoretical model explained team performance improvement more than twice as well (R^2^=40.6%) than prior related research (R^2^=18.5%).

**Conclusions:**

The focused immersion caused by team video gaming increased team performance while the enjoyment component of flow decreased team performance on the posttest. Both flow and team cohesion contributed to team performance, with flow contributing more than cohesion. Team video gaming did not increase team cohesion, so team video gaming effects are independent of cohesion. Team video gaming is a valid practical method for developing and improving newly formed teams.

## Introduction

Important organizational work is often performed in teams. Therefore, effective teamwork has long been a critical area for research [1,2]. Yet, evidence continues to emerge that shows teamwork is often ineffective. For example, one practitioner survey found that 97% of employees and executives believe that team alignment is critical to their effectiveness, yet 86% blame a lack of effective team collaboration for workplace failures [3]. As a result, although a strong body of research exists on effective teamwork, there is continual need for research on how to build productive teams.

A common thread through prior research is that effective teamwork requires interpersonal skills [4], the development of effective ways to share work, and team members’ willingness to exert effort toward the team task [5]. To help teams develop these critical skills and norms, some organizations employ team-building activities. Meta-analyses show that team-building activities significantly increase team performance and productivity [6]. And given that employee disengagement costs the US economy as much as US $350 billion every year [7], some organizations invest extensively in team-building activities. But team-building activities used by organizations can be both time-consuming and expensive. Ropes courses and other challenge retreats are common team-building interventions that have been moderately effective in increasing team performance [8]. But these interventions take employees away from work for a day or longer. The time and expense required by many team-building interventions have led some to argue that team building is a waste of resources [9]. This raises an important question and opportunity for research. Can effective team-building interventions be created that increase team performance but take less time? This study examines team video gaming (TVG) as a possible solution.

For many people, video games have become a pervasive part of life. In the year 2020, 2.6 billion people worldwide played video games [10]. Research on online games shows that players are drawn to games for opportunities for achievement, entertainment, immersive experiences, and interactions with other players [11]. Not all video games are designed exclusively for fun, entertainment, and escape [12]. Video games can use entertainment and enjoyment as a means to help accomplish a variety of worthwhile objectives, such as education [13], training [14], gaining experience in complex situations [15], and facilitating social networks [16].

**Figure 1:**
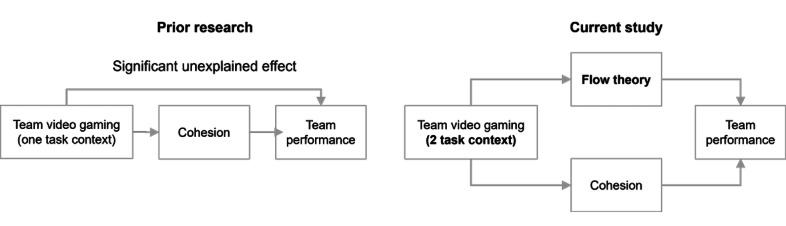
Summary of study contributions.

TVG gives players practice at forming and working effectively with other players in teams [17]. Players work together against an opponent to achieve a common goal. Video games such as Halo, a competitive and cooperative first-person shooter game, and Rock Band, a cooperative music game, encourage players to collaborate and engage as a team to successfully complete shared objectives. Much like competitive sports, teams playing TVG exhibit player engagement, team communication, and strategy formation to achieve a common goal [18]. Another reason TVG is a candidate for team building is that some research suggests that TVG improves team cohesion [19]. Cohesive teams developed social relationships and trust, attraction to the team and to the team tasks, and a knowledge of how to work together [20,21]. This improves team performance on subsequent tasks [22-24]. Some organizations provide video game lounges containing gaming equipment because employees enjoy playing together [25]. However, only one academic study has examined the use of TVG for team building and measured subsequent team performance. Keith et al [26] compared the effects of team building with and without TVG on subsequent team performance in a single task context. They found that teams using TVG increased team cohesion and increased subsequent team performance. However, there was a direct positive effect of TVG on team performance that was not explained by team cohesion. Our study found that TVG also promotes team flow, which accounts for the previously unexplained increase in team productivity. Thus, it provides a better explanation of how team building with TVG improves team performance. This study also found that TVG improved team performance in 2 different task contexts. Figure 1 reflects how the current study compares with that of the study by Keith et al [26] (labeled as “Prior research”) and the unique contributions of our study in bold.

We draw from gamification theory to help explain how TVG can help teams become more productive. *Gamification* is the practice of using game-design elements (ie, rules, goals, interactions, rewards) in nongaming contexts [27,28] or making processes more “game-like” [29]. An important goal of gamification is to promote player engagement [30] so the player is fully involved or immersed in a physical or mental activity [31]. This gamification perspective motivated our selection of *flow* theory to enhance the team cohesion model by Keith et al [26] of TVG-driven team performance. *Flow* theory explains the psychological state of being fully immersed in, and focused on, a task [12,30,32,33]. Research has shown that engagement on work tasks can be manipulated and that a team’s collective engagement on one task can recur on subsequent tasks [34-36]. Therefore, we expect teams that experience flow during TVG will also experience flow on subsequent team tasks. To promote this state of flow, we had new teams play commercially available team video games with which they were already familiar, which benefits the team-building process. There are some unique aspects of team cohesion and flow theory that are relevant to the TVG context, thus necessitating both theories. Our research questions are (RQ1) “Can gamifying the team building process via TVG be an effective strategy to improve team performance?” and (RQ2**)** **“**Can team flow explain the effects of TVG on team performance above and beyond that of team cohesion alone?”

To test our theoretical model, we implemented TVG as a treatment in a laboratory experiment in 2 different contexts or tasks. For the first task, we used the same geocaching task for the pretest and posttest used by Keith et al [26]. For the second task, we used a completely different tower-building task for the pretest and posttest. Using a second task that is distinctly different from the first study allowed us to demonstrate that the effects of the TVG were not limited to 1 task. Our research focuses on helping new teams become productive. Such teams are different from long-term, existing teams because the team members have few, if any, prior relationships among team members. New teams may persist for long or short durations. Helping newly formed, short-term teams become productive is important because many teams in organizations are formed for short durations [37] or may include temporary employees [38].

### Theory: How Team Video Gaming Improves Team Performance

#### Team Building With Video Gaming

Games have long been practiced as a strategy for team building. Games and challenges are used in many traditional team building interventions (eg, ropes courses, competitive team games). The video games we used were developed for entertainment purposes and not for team building but can be used to effectively accomplish the goals of team building. Research on team building has revealed 4 fundamental components of team-building interventions: goal setting, interpersonal relations, problem solving, and role clarification [39-42]. These components are not necessarily activated equally in every team-building intervention. A team-building intervention might include one, multiple, or all these components in varying degrees. Although specific team video games differ in the degree of support for all 4 team-building interventions, some TVG can activate all 4. While playing team video games, individuals increase their skill and share knowledge with teammates [43]. Individuals learn to depend on and be dependable for other team members, which results in building relationships and strengthening teamwork skills [44]. Moreover, the team attributes developed during this process can and do carry over into their cooperative work in high-tech, cross-functional, team-centered workplaces [6,26,45,46]. Our first hypothesis will test this theory: (H1) Teams using TVG will perform better on subsequent team tasks than teams who do not use TVG.

While this finding alone is useful, it is even more important to understand why TVG has this effect. In the following sections, we review 2 theories that may explain the effect of TVG.

#### Team Cohesion

Team cohesion theory is the dominant historical explanation of how team-building interventions increase team performance (eg, [22,47-49]). At a high level, team cohesion theory states that teams should develop the following attributes to be able to work effectively together: (1) social relationships and trust, (2) attraction to the team, (3) general attraction to team tasks, and (4) knowledge of how to work together [20,21]. Traditional team-building interventions that do not use team video games create team cohesion that subsequently carries over into other tasks, thus improving team performance [22-24].

Team flow is often the “go-to” theory used to explain team performance. However, prior research shows that team cohesion theory has only limited capability to explain the team performance effect that comes from video gaming [26]. Therefore, we turn to flow theory, which is more appropriate for video game designs.

#### Flow

*Flow state* refers to the psychological condition of being totally immersed in, and focused on, a task [12,30,32]. The concept of flow has been used to explain the deep engagement of individual persons in activities involving high levels of intrinsic motivation [50] and the optimal state of engagement [12,30]. Flow is typically measured using 5 subconstructs: focused immersion, heightened enjoyment, control, curiosity, and time dissociation.

When individuals enter a state of flow, they feel like they are in control, their curiosity is engaged, and they enjoy themselves [51]. They focus intently on the task at hand and tune out outside stimuli. When experienced by a team (eg, [52,53]), these aspects of engagement may promote team performance—because the team is curiously engaged, feels in control, focuses intently on their task, and tunes out distractions, all while having a good time.

Video games are known to induce flow states easily because they meet 3 necessary preconditions [50]. First, to experience flow, there must be a clear goal to be achieved. Playing video games typically has a superordinate goal of winning the game or performing well. Users must perform tasks that support achieving the superordinate goal. Second, there must be feedback that reflects the degree of performance toward the goal [54]. The games used in this research provide clear, real-time feedback to teams through dashboards that show players how well they are performing in real time. As the games progress, teams can see where they stand and can focus their efforts accordingly. Finally, and perhaps most critically, there must be a balance between skill and the level of “appropriate challenge” in the task of interest [50,54-58]. Flow can be achieved only if the challenge is appropriately matched with skill. In other words, the task cannot be either too easy or too difficult. In the TVG context, playing against a much more skilled team would result in frustration, whereas playing against a much less skilled team would be boring. Thus, video games do not drive flow; rather, a state of flow occurs when using video games provides an appropriate challenge. (H2) TVG will increase the degree of perceived appropriate challenge in a subsequent task. (H3) Appropriate challenge has a positive effect on flow.

### Flow and Performance

**Figure 2:**
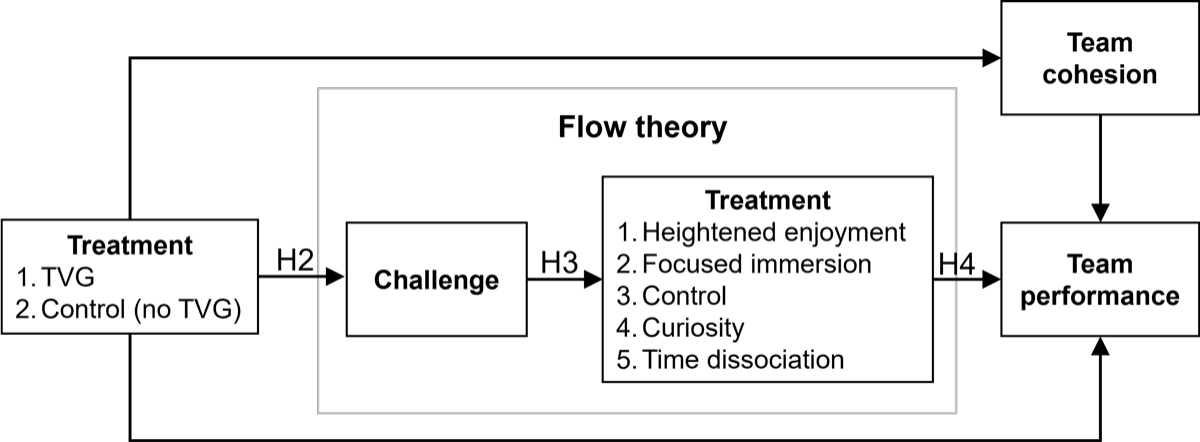
Theoretical model. TVG: team video gaming.

The positive effects of flow have been observed in prior research [59-61]. As flow increases, team performance should increase [52,53] because flow represents deep engagement and focus on the task at hand [62]. The strongest outcome of flow in the model by Agarwal and Karahanna [62] was the effect on perceived ease of use. Such a finding suggests that when we experience flow, we perceive the task at hand to be easier to accomplish. Furthermore, Rutkowski et al [63] found that *focused immersion* (a subdimension of flow) led to greater performance in virtual teams. When teams are immersed in a task, they ignore external (distracting) stimuli that may divert attention away from the task [12]. Thus, an increase in flow should increase performance (Figure 2). (H4) Flow, including (1) focused immersion, (2) heightened enjoyment, (3) control, (4) curiosity, and (5) time dissociation will increase team performance.

## Methods

### Participants

We used a laboratory experiment design to test our theoretical model. Participants in this study were undergraduate students at a private western university in the United States. They were randomly assigned to teams of 4 participants, and teams were randomly assigned to treatments. However, we questioned the participants prior to making their final team assignment to detect whether they had any preexisting relationships with potential teammates. If they did, the teams were rearranged to minimize the impact of prior relationships. Participants assigned to TVG treatments could select 1 of 2 popular games based on their familiarity with the game. Subjects chose either Rock Band 3 or Halo 4. The popularity of these games makes it likely that students have played them before. These games have also been used in past research [16,64]. This allowed team members to play the game for which they had the most experience and that they found most interesting and engaging. This should have maximized the likelihood of the video game effect and how a team would select a video game in real life. To control for video game ability (and thereby balance appropriate challenge and skill), we asked participants about their level of experience with the game and then balanced the level of video game experience across teams so that teams were competing against other teams of roughly equivalent skill. A total of 586 individuals were divided across 155 teams. However, only 444 participants completed all survey responses measuring latent constructs used to estimate the theoretical model. A few teams had only 3 participants due to no-shows at the lab. Comparative tests showed few differences between 3-person and 4-person teams as noted in the Results section. Of those who chose to report, 141 (141/583, 24.2%) were female, 469 (469/583, 80.4%) were Caucasian, 59 (59/583, 10.1%) were Asian, and 41 (41/583, 7.0%) were Hispanic. The average age of participants was 22.9 years.

### Study Design

The study involved a (1) pretest of team task performance to establish a baseline, (2) treatment, and (3) posttest of team task performance, to measure performance improvement.

#### Task

The tasks in the experiment were designed to replicate the context of a newly formed work team under time pressure. Therefore, the task met the following criteria: (1) it was time sensitive—there was a limited amount of time available to complete the task; (2) it had objective performance measures that were readily calculable—this allowed teams to evaluate their own performance and compare their performance to other teams; (3) the teams selected their own strategies and division of labor—this allowed team members to benefit from their own creativity and ingenuity; and (4) the task required team members to coordinate and collaborate to achieve the best results. We implemented 2 distinct tasks that allowed us to collect objective measures of team performance. Using 2 different tasks allowed us to measure the influence of the tasks on the results.

*Task 1* was based on a mobile application called “Findamine” (pronounced “find a mine”) that was created for research purposes and has been successfully used in prior field experiments in information system (IS) research [26,65], but with modifications for our context. Findamine is a geocaching mobile application that generates clues for finding specific landmarks. Rather than giving GPS-based latitude/longitude coordinates, the application gives players short, text-based clues (eg, “This statue depicts the founder of this university.”) that help participants identity the specific location. The destination locations were distributed across the large campus. Teams earned points by successfully deciphering the clue, travelling to the location, and taking a picture of themselves at the location. The pictures of subjects in front of the landmark were automatically uploaded through the mobile application.

Participants could identify and visit more locations by dividing into pairs. So, division of labor, communication between the team members, and collaboration were rewarded. However, the application tracks the total time elapsed from opening a clue until the correct picture (verified by the GPS coordinates embedded in the photo) is submitted through the application. The natural log of the minutes elapsed was deducted from the possible clue points to reward teams for the speed of their performance in addition to accuracy.

At the conclusion of the task, teams returned to the start location where their performance was displayed in a “leaderboard” so that they could compare their results with those of other teams. In summary, this task enabled all 3 preconditions for flow, namely (1) a clear goal, (2) performance feedback, and most importantly, (3) a challenge that is commensurate with their skills [50,55]. However, given that this geocaching task is somewhat “game-like” itself and may be correlated with the characteristics of TVG, we created a second task (teams only completed 1 of the 2 tasks).

*Task 2* was drawn from prior laboratory research on team tasks and performance [66]. It included a timed task of building a tower out of dry uncooked spaghetti noodles and marshmallows. However, there was no leaderboard or real-time feedback about how they were performing relative to other teams, thus minimizing the competitive element. Like the geocaching task, participants were divided into teams of 4 (while minimizing the likelihood of prior relationships among team members). Teams were given 7 minutes to build the tallest tower possible that would remain standing. Performance was measured as the height in inches of the tower. Table 1 shows the number of teams in each treatment assigned to each task.

**Table 1:** Sample size: number of teams for each treatment and task.

Treatment	Task 1 (geocaching app)				Task 2 (tower building)		
	Number of participants	Number of teams	Number of participants per team, mean	Number of participants		Number of teams	Number of participants per team, mean
Control	191	51	3.75	147		38	3.87
Team video games	112	30	3.73	136		36	3.78

After all data were collected, z scores were calculated for both Task 1 and 2 performances in order to make the results comparable. We also included *task type* (Task 1 coded as 1 and Task 2 as 0) as a covariate in our hypothesis testing.

After being assigned to teams, participants performed either Task 1 or Task 2 (depending on the date of the study) as a baseline measurement of team performance. Similar to prior research [26], those assigned to Task 1 (geocaching) downloaded the app on only 2 of the phones possessed by team members. The app had 6 clues ready with a 25-minute time limit. The team’s total score was the combined total of the points on both phones. The phones of the other team members could still be used for communication. Teams were given 5 minutes to plan a strategy. Immediately at the 5-minute mark, the 25-minute timer began during which they could find the clues. As an incentive, teams were notified that the highest-scoring team from each day’s participants would earn US $20 Visa cash cards for each member. As locations were found, the results were loaded into a website leaderboard in real time. Upon returning, each team was shown their standing on the leaderboard, and their performance was recorded.

Teams assigned to Task 2 (tower) were placed in an isolated room that was not visible to any other participant teams. They were given a standard number of marshmallows and dry spaghetti noodles. They were also given a brief review of the rules: (1) build free-standing towers not adhered to any furniture or walls, (2) no use of smartphones for ideas or tips, and (3) total score is the combined height of the 2 tallest towers (to give teams the opportunity to determine how to divide roles). Finally, a 7-minute timer was started and left in the room with the team. Similar to Task 1, participants were notified beforehand that the highest-scoring team of that day would earn US $20 Visa gift cards.

#### Team Intervention: Treatments

Upon completing the pretest, teams were randomly assigned to 1 of 2 treatments: (1) TVG or (2) control (no TVG). Those assigned to the *control* treatment were asked to spend the next 45 minutes by themselves. Team members were instructed to not speak with each other until the posttest began. This was intended to replicate the practical context where no team building occurs. However, they were left at liberty to work individually on homework or any other pursuit unrelated to the experiment.

**Figure 3:**
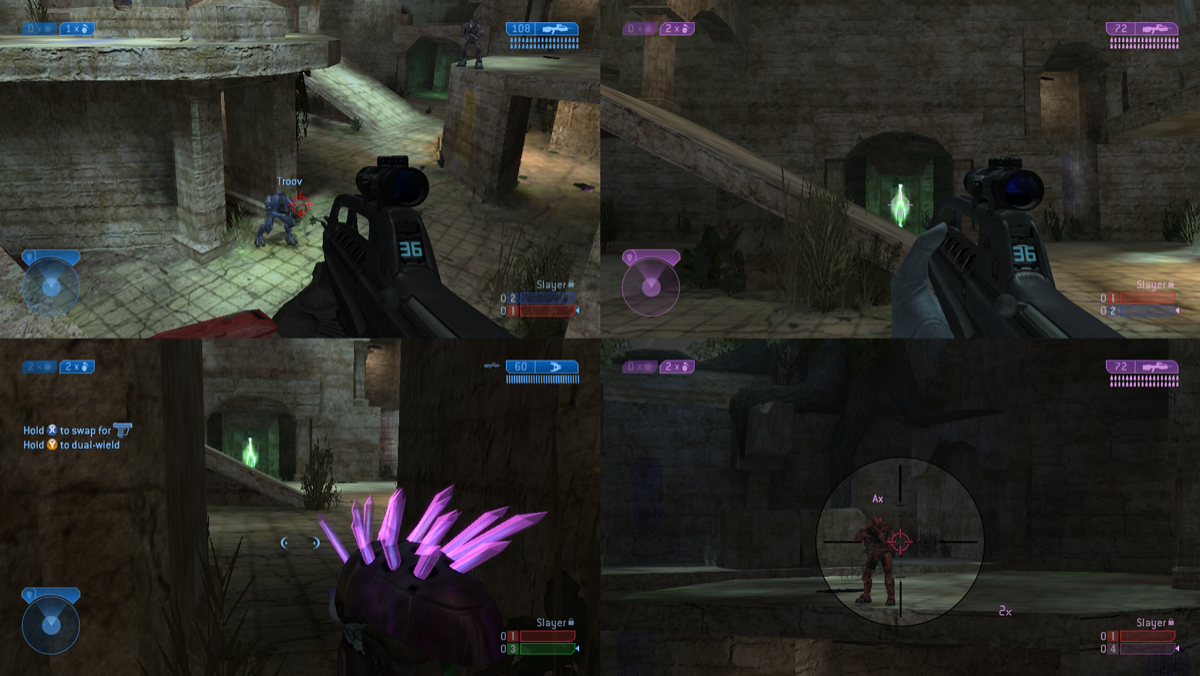
Screenshot of Halo.

**Figure 4:**
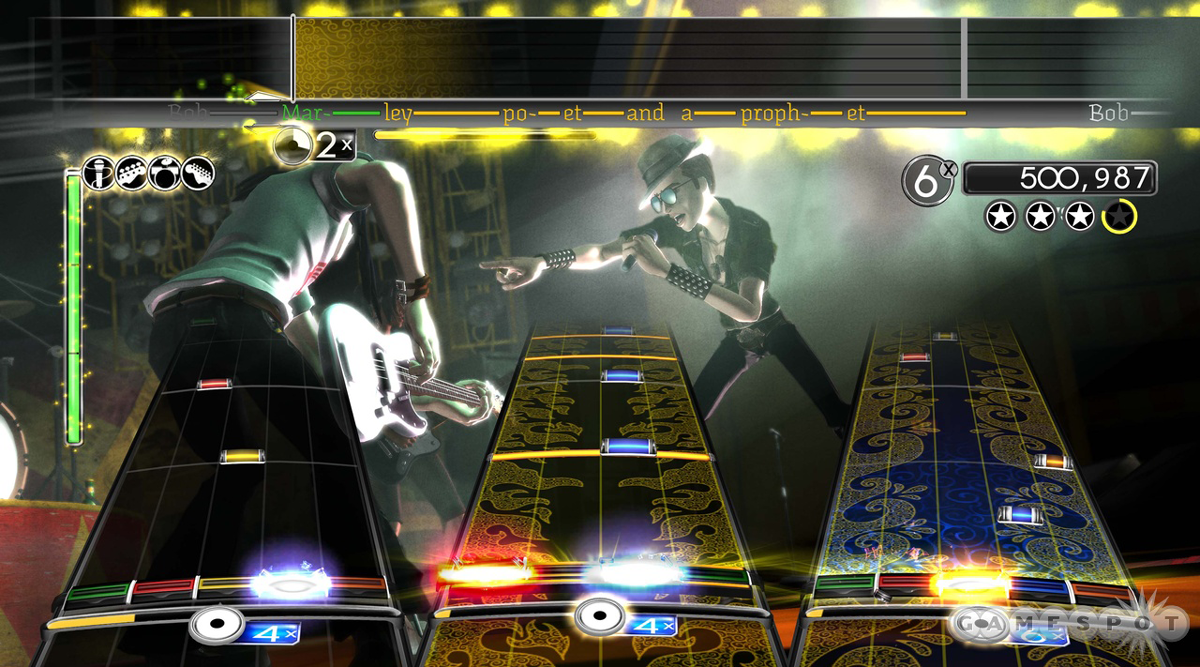
Screenshot of Rock Band.

In the *TVG* treatment, participants played Rock Band 3 or Halo 4. These 2 games were selected primarily because of the interdependent nature of the team tasks. In Rock Band, the players must coordinate their activities to perform the songs correctly. In Halo, the players must coordinate their attacks and defensive strategies to beat the other team. Teams in the Rock Band condition were tasked to earn the highest possible score across any 4 songs of their choosing. The team that earned the highest score earned large candy bars for each member. Those in the Halo 4 condition played 3 rounds of the team-based “capture the flag” subgame against the other team in their cohort. The team winning at least 2 out of 3 matches earned large candy bars. Both treatments lasted 45 minutes. Figures 3 and 4 visualize the gameplay of both games.

Importantly, those in the TVG treatment were not simply left to themselves. Rather, they were playing in a cooperative-competitive environment in which the team they were competing against was in a nearby, but separate, room. A facilitator was assigned to handle technical problems and ensure that the teams played the games according to instructions and fully participated.

#### Posttask: Measuring Change in Team Performance

After the treatment, teams were again assigned to complete another round of the same pretest task so that team performance could be measured as the relative percent improvement from the pretest. For Task 1 (geocaching), the study administrators downloaded 7 new clues for new locations on campus to the 2 phones for each team running the application. The increase from 6 to 7 clues in Task 2 was because pilot tests revealed that participants gained experience and skill from Task 1 that translated into faster task completion times. Therefore, to even out the total time required by Task 1 and Task 2, the number of clues was increased for Task 2. Once again, the teams had 5 minutes to strategize and then 25 minutes to find and photograph themselves at as many of the locations as possible. Upon finishing this task, the teams viewed their standing on the leaderboard and completed another survey measuring flow and the other key variables. For Task 2 (tower), participants were placed back into the same room with a fresh set of spaghetti and marshmallows and a clean workspace. They were given another 7-minute timer and set to work.

After the task, each team member took a survey measuring flow and several other covariates. It is important to note that the survey measures referred to the participants’ flow state during the geocaching or tower-building task—not the TVG treatment. This is significant because an important assumption of our research is that achieving a flow state during a team intervention (ie, TVG) can increase the likelihood of entering a state of flow on subsequent team tasks as with the geocaching task.

### Measures

The attitudinal variables in this study were measured using latent construct items drawn from prior research and adapted for this study. Team flow was adapted from the construct of cognitive absorption [62]. It is an aggregate, second-order construct [67] that includes the subdimensions of *focused immersion* (FI), *heightened enjoyment* (HE), *control* (CO), *curiosity* (CU), and *time dissociation* (TD) [62]. Based on conceptualizations from flow theory [50], 3 items measuring *appropriate challenge* (CH)—or how well the task was equal to (not beneath or above) the skill level of the team—were created new for this study. They were first pilot-tested and met all criteria for validity and reliability (as demonstrated in the following sections). As aforementioned, team performance was measured as the team composite percent increase over the score earned in the pretest task.

Team cohesion was measured based on the conceptual model by Carron et al [68]. Although there are 4 primary subcomponents of team cohesion, the 2 most relevant to the short time duration of our TVG treatment are *group integration-task* (GI-T) and the *individual attractions to the group-task* (ATG-T). The variables Group Integration-Social (GI-S) and Individual Attractions to the Group-Social (ATG-S) were eliminated from the study because we found that the items demonstrated very poor validity and reliability despite being drawn directly from prior research. We believe this indicates the procedures did not provide enough time for the social constructs measured by these instruments to develop to the point where they could play a role in our results. Team flow and team cohesion were modeled as first-order reflective and second-order formative constructs based on prior research [62,68]. *Task interdependence* was included as a control variable because it is a known potential confound in team cohesion research [69]. Items were adapted from Sharma and Yetton [70].

In addition, we included 2 controls: *task type* (geocaching or tower-building) to control for task-related performance differences and their score on the pretest baseline task (standardized to a z score). The latter control is important because teams in both tasks may have been able to achieve a “ceiling” effect where, if they performed extremely well on their pretest task, they would have less room for improvement on the posttest task.

### Measurement Model Testing

Pre-analysis was performed to test the convergent and discriminant validity of the reflective subdimension measures, test for multicollinearity, and ensure reliabilities. The constructs, question wording, and outer loadings are summarized in Multimedia Appendix 1 (Table A1). As indicated in Tables A2 and A3 in Multimedia Appendix 1, after removing HE4, CO2, ATG-T3, and ATG-T5, all validity criteria were met. In particular, all average variances extracted (AVEs) were above the 0.50 recommended cutoff and greater than the squared correlation between the focal construct and the subdimensions [71]. Composite reliability was greater than 0.70 for every subdimension. Concerning discriminant validity, the cross-loading matrix in Multimedia Appendix 1 (Table A3) reveals that all item loadings were greater than their cross-loadings. However, the validity scores (Cronbach alpha) for ATGT and CO were slightly below the 0.70 threshold (see Multimedia Appendix 1, Table A2) [72]. Because ATGT is a well-validated instrument, we retained the items as indicated. CO likely had minor problems because only 2 items remained to measure it [73]. However, as all other reliability criteria were met, we continued with the analysis.

We also tested for reliability and validity at the second-order factor level for flow and team cohesion (see Multimedia Appendix 1, Table A4). To test these constructs, latent factor scores were first generated for the subconstructs of both flow (HE, CO, CU, FI, TD) and team cohesion (ATG-T and GIT). These latent factor scores were then used as reflective indicators for the second-order factors. In summary, after testing the measurement model at the highest-level of each construct, all criteria for convergent validity, discriminant validity, and reliability were met. In addition, multicollinearity was not a significant concern as all variance inflation factor scores were below the recommended maximal cutoff of 10 [73]. Therefore, we opted to keep the remaining items and proceed with the hypothesis testing. Overall, the results indicated acceptable factorial validity and minimal multicollinearity or common method bias based on the standards for IS research [74].

### Team-Level Constructs

In this study, our treatments were administered to teams, our hypotheses are at the team level, and we predict team-level performance. Thus, the team is our unit of analysis. These perceptions are what Klein and Kozlowski [75] refer to as “emergent unit properties” because they “originate in experiences, attitudes, perceptions, cognitions, or behaviors that are held in common by team members.” The team-level attitudinal measures reported in this research were aggregated as means of individual scores of team members. We used the *direct consensus* composition model for the constructs of challenge, components of team flow, and group interaction. We used the *referent shift* composition model for attraction to the group [76]. Multimedia Appendix 2 describes the approach we used to justify the aggregation of individual-level responses into team-level measures.

### Comparison of Productivity Increase Across Treatments

To examine changes in team productivity between the pretest and posttest in the control treatment versus the video game treatment, we calculated the percent productivity change for each team and used a one-sided *t* test to compare average changes in productivity across the treatments.

### Validation of Theoretical Model

We validated our flow-based theoretical model using the survey responses analyzed in 2 path models using the partial least squares (PLS) structural equation modeling technique in SmartPLS 3.2.6 [77]. Use of this analytical approach was appropriate because PLS does not depend on normal distributions or interval scales [78] making it ideal for our objective measures of task performance. The *t* statistics were generated from running 10,000 bootstrap procedures. We estimated 2 models. In the first model, we combined all aspects of flow into one flow measure so we could measure the effects of challenge on flow taken as a whole and how flow impacts team performance. In the second model, we estimated the effects of challenge on the subcomponents of flow and the effects of the subcomponents of flow on performance to examine the differential role of components of flow as suggested in prior research [12]. Both models were based on team-level scores.

## Results

### Comparison of Productivity Increase Across Treatments

Table 2 depicts the percentage increase from pretest to posttest for the work tasks. For both tasks, teams in the TVG treatments increased their posttest performance over their pretest performance more than the teams in the control treatment. The TVG treatment in Task 1, geocaching, resulted in a 49.6% average improvement compared with 20.3% for the control teams, for a difference of 29.3% (*t* test, *P*<.001). The tower-building task (Task 2) produced greater variance in term performance than Task 1 and a clear “ceiling” effect—meaning that teams who built a very tall tower during the pretest were not able to improve their score by as great a percentage as the geocaching task. Despite this, the TVG treatment resulted in a 72.1% average improvement compared with 49.5% improvement for the control teams, for a difference of 22.6%.

**Table 2:** Percent change in team productivity from the pretest to posttest for Tasks 1 and 2.

Task	Control treatment mean (SD)	Team video games treatment mean (SD)	Percent difference, (%)
Task 1 (geocaching app)	20.3 (.328)	49.6 (.399)	29.3
Task 2 (tower building)	49.5 (.754)	72.1 (.801)	22.6

An analysis of variance controlling for game (Halo or Rock Band), gender (percent female), age, team size, and task showed that the TVG treatment had a very strong and significant effect on improving team performance (*F*_1_=8.760, *P*=.004). Thus, H1, which predicted that teams using TVG would perform better on subsequent team tasks than teams who did not use TVG, was supported for both tasks. Additionally, we examined differences in mean scores by video game, task, gender, and team size; these are described in Multimedia Appendix 3.

### Validation of Theoretical Model

**Figure 5:**
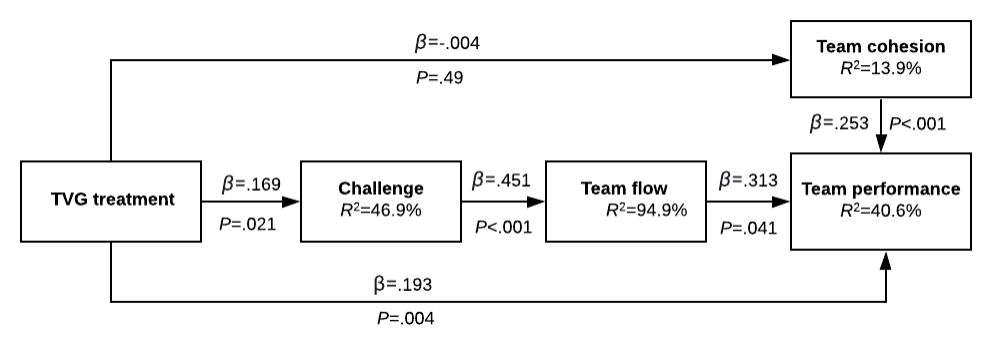
Hypothesis testing. TVG: team video gaming.

With H1 supported, our next task was to validate our theoretical model explaining why TVG has a strong positive effect on team performance. Figure 5 shows the path coefficients (*β*) above each relationship and *P* values in parentheses based on a team-level analysis. R^2^ values are shown for the endogenous variables. Although not depicted in Figure 5, task type, gender (percent female), and team size were included as covariates explaining each endogenous variable.

Supporting H2, the TVG treatment increased the teams’ perceptions of the challenging nature of the subsequent tasks (*β*=.169, *P*=.021). As predicted by H3, challenge increased the perception of team flow (*β*=.451, *P*<.001). The exceptionally high R^2^ for team flow (94.9%) is not unexpected since, as noted earlier, challenge is an essential prerequisite for flow. Perhaps most central to our study, H4 was supported, in that team flow significantly increased performance (*β*=.313, *P*=.041). The control variables gender (*β*=–.056, *P*=.21), team size (*β*=.017, *P*=.40), and task type (geocaching versus tower building; *β*=.265, *P*=.20) had no significant impact on team performance.

Although not specifically hypothesized, we re-examined the relationships tested in prior research on team cohesion. TVG did not significantly increase team cohesion after controlling for task type (*β*=–.004, *P*=.49). Team cohesion did increase team performance (*β*=.253, *P*<.001).

**Figure 6:**
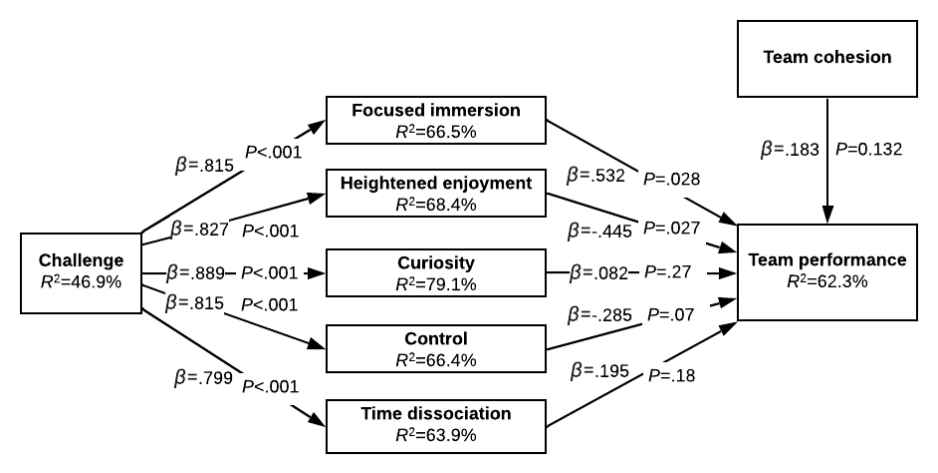
First-order team flow.

With hypothesis testing completed in Figure 5, Figure 6 provides a deeper understanding of team flow by depicting the results of the model when the subcomponents of flow are estimated as opposed to the second-order flow factor. This allows us to better compare the effects of flow versus team cohesion. The treatment was removed from this model for simplicity. Challenge contributed positively and significantly to all components of flow (*P*<.001). However, the effects of the components of flow on performance were much more differentiated. Focused immersion positively influenced team performance (*β*=.532, *P*=.028). Surprisingly, heightened enjoyment had a significant negative effect on team performance (*β*=–.445, *P=*.027). Control had a moderately significant negative effect (*β*=–.285, *P=*.07). Curiosity (*β*=.082, *P=*.27) and time dissociation (*β*=.195, *P*=.18) had no significant effects on performance. Breaking flow into its subconstructs produced a model that better explains the effects of a TVG treatment on performance. The R2 value for team performance improved from 40.6% in Figure 5 to 62.3% in Figure 6.

Although not depicted in Table 2 and Figures 5 and 6, there were significant effects of the type of task performed (1=geocaching versus 0=tower-building): After controlling for all other relevant paths, those who participated in the geocaching task experienced a greater sense of appropriate challenge (*β*=.739, *P*<.001) and thus, greater flow (*β*=.613, *P*<.001) while developing less team cohesion (*β*=–.375, *P*<.001). In addition, as expected, higher performances in the pretest led to lower relative increases in posttest performance (*β*=–.539, *P*<.001).

Finally, the control variables gender (*β*=–.082, *P*=.12), team size (*β*=.004, *P*=.48), and study (geocaching versus tower building; *β*=–.181, *P*=.36) had no significant impact on team performance. Table 3 summarizes the hypotheses and main results of this study.

**Table 3:** Summary of hypotheses and findings.

Hypothesis number	Support	Finding
H1	Yes	Team video gaming increased team performance on subsequent team tasks.
H2	Yes	Team video gaming increased perceived appropriate challenge in subsequent team tasks.
H3	Yes	Appropriate challenge increased flow.
H4	Yes	Flow, comprised of all flow components, increased team performance.
H4.1	Yes	*Focused immersion* increased team performance.
H4.2	No	*Heightened enjoyment* decreased team performance.
H4.3	No	*Curiosity* did not increase team performance.
H4.4	No	*Control* did not increase team performance.
H4.5	No	*Time dissociation* did not increase team performance.

## Discussion

### Principal Findings

In response to our 2 research questions, the primary findings of this research are first that the team-building process can be implemented with TVG. Unlike traditional team-building exercises, which focus the attention of team members on specific team-building objectives, these objectives are met naturally as the teams focus on cooperating to play the game. Existing features in the games used in this research supported cooperation and competition through enjoyable and challenging game scenarios, thereby effectively accomplishing the team-building process. Second, teams that played TVG experienced greater flow and exhibited greater performance on subsequent team tasks. This effect was found with 2 different video games and with 2 different work tasks; thus, the positive effects of team building with TVG are not limited to 1 video game or 1 work task. TVG provides some benefits traditionally attributed to team cohesion: A limited form of team attraction, attraction to the task, and roles and scenarios embodied in the game serve to facilitate effective division of labor and cooperation within the team. But TVG does not manipulate team cohesion. The effects of team cohesion on team performance are independent of TVG. Team cohesion has a small, positive impact on team performance but is not affected by TVG. Instead, TVG improves performance by increasing appropriate challenge, which increases flow. The positive effect of flow on performance is stronger than the positive effect of team cohesion on performance. Focused immersion is the component on flow that increases team performance. Heightened enjoyment is a component of flow that decreases team performance. These findings constitute a better explanation of how TVG increases team performance than prior research. We found a much stronger effect size for team performance. In particular, our model explained 62.3% of the variance (R^2^) in team performance across 2 distinct types of team tasks compared with the 18.5% explained by Keith et al [26].

### Implications

This study contributes to the team-building literature in a unique way. We demonstrated that TVG is an effective team-building strategy. TVG does this by creating an immersive environment that motivates team members to rise to a challenge and achieve a flow state. Most importantly, much like the concept of work engagement state [79], this research demonstrated that flow state can be carried over to improve performance on subsequent team tasks.

One important implication of this research is that the clarity and explanatory power of our model improved substantially when we analyzed the components of flow at the first-order subconstruct level. Given the adequate measurement model metrics we found for flow as a second-order construct, one could reasonably expect that each subconstruct of flow would have an important and positive effect on team performance. However, that was clearly not the case in our data. The positive flow effect came entirely from focused immersion. The focused immersion aspect of flow experienced during TVG carried over into subsequent tasks. That is not the case with the other subconstructs. Enjoyment had a negative effect on the subsequent task performance, whereas curiosity, control, and time dissociation produced no significant effects on team performance.

These noneffects can likely be attributed to the specific attributes of our experimental tasks. Curiosity requires time for contemplation, but time was limited during both the geocaching and tower-building tasks. Further, teams were competing against other teams and had limited time and could not see the scores of the other teams until after they completed the task. This likely explains why they felt little control. Lastly, because our tasks were short and time was controlled and limited, the teams had to keep track of the time they had left while completing the pretest and posttest tasks. Thus, they could not experience time dissociation. These aspects of flow might be experienced in team tasks that are less time constrained.

However, the time limitations of our tasks do not account for the significant *negative* effect of heightened enjoyment on team performance improvements (*β*=–.445, *P*=.027). Any heightened enjoyment developed during TVG may have led to greater heightened enjoyment of subsequent tasks, which led to lower performance on those tasks. This effect is somewhat harder to explain, particularly considering that prior research has found that heightened enjoyment is positively correlated with an employee’s absorption in work tasks and motivation [46] and even work teams’ collective efficacy beliefs [36]. The contradiction of our findings with prior research may be explained by the source of enjoyment that comes from video games versus work fulfillment. For example, the enjoyment that comes from work accomplishment may be different from the enjoyment from leisure experiences [80]. Therefore, while TVG may help build team focus, it may also create a “let down” effect where the type of enjoyment built during TVG is incongruent with the type of enjoyment that comes from accomplishing work tasks. Future research should explore this possible negative effect of enjoyment on team tasks in the workplace. If this negative effect persists, future research could also examine whether there are types of team video games that produce a form of heightened enjoyment that is more in line with work fulfillment and work enjoyment.

There are other reasons future research should measure and analyze flow at the subconstruct level. For example, the influence of challenge on flow was somewhat suppressed when flow was measured as a second-order construct (*β*=.451, *P*<.001) but was substantially stronger, ranging from .799 to .889, for the individual subconstructs. Another consequence of assessing flow at the aggregate was to understate the impact of flow on performance and to overstate the positive influence of team cohesion on performance. Our results also suggest that flow can be measured and assessed at the team level by aggregating responses of individuals when members of teams receive the same treatments and there is a high degree of reliability in terms of how team members rate the constructs [52,53,81]. These criteria were met in this research.

There are many additional research applications in which the effects of team flow could be examined. For example, our results demonstrate an effect of team flow in a controlled, laboratory environment; however, team flow could also be adapted to information technology (IT) project teams and subteams to see how time-critical IT projects can be enhanced with video gaming at a project kickoff meeting.

An important implication of these findings is that video games manipulated flow but not team cohesion and that flow had a stronger impact on performance than cohesion. Team cohesion has received decades of research support in a variety of settings, and we do not claim flow will be more important to performance than cohesion for all settings. Flow is relevant in the TVG context where the task requires focused immersion, heightened enjoyment, control, curiosity, and time dissociation. While some work tasks meet these criteria, some do not. It remains to be seen whether team building with TVG will benefit these other tasks.

Furthermore, both flow and team cohesion matter. Both theories help explain team performance, but each explains different aspects of team building. Team cohesion explains the social integration of the team and their attraction to the task at hand [20] whereas flow explains the state the team is in, the *manner* in which a task is performed. We believe the type of task will likely determine which factor provides the dominant explanation. Flow exerted a stronger influence in our context—newly formed work teams completing time-sensitive, short-term tasks. Cohesion will likely exert greater influence on performance when there is time to develop relationships with team members and an attraction to the task. Future research might extend our results by discovering the boundaries between task types that explain where one theory might be dominant over the other—although both are likely to be relevant to some degree in every task.

The overall effect of TVG to increase team performance was significant for both tasks used in this study: 29.2% for Task 1 and 22.5% for Task 2. Thus, they exceeded the increase of 20% found by Keith et al [26]. This means if tasks performed in our study are representative of other tasks, team building for 45 minutes with TVG for newly formed work teams may increase performance for subsequent tasks requiring several hours or more. TVG requires much less cost and time than traditional team-building activities like retreats and ropes courses. However, it should be noted that the teams in our study were highly engaged in the TVG task.

The finding that the geocaching task produced less team cohesion may be the result of the teams splitting up to find different landmarks, whereas all team members worked together on the tower-building task. Plus, geocaching teams had to determine what each landmark was, determine where it was located, and go to that landmark to take a picture there, so the geocaching task was more challenging and produced more flow than the other task. The descriptive summary in Multimedia Appendix 1 (Table A7) also has some interesting implications. For example, Halo appeared to induce greater improvements in team performance than Rock Band but did not produce greater flow. Therefore, there may be alternative explanations to explain the difference between game features that lead to performance differences. And although Halo led to greater team performance, women were more likely to select Rock Band. It is also interesting that women found more heightened enjoyment in the posttest in our study. Since women were the minority participants, this effect may have come from being part of teams that were more diverse demographically. Future research should explore how team homophily moderates the TVG effect.

Lastly, the strong and consistent impact of appropriate challenge on each subdimension of flow provides strong empirical evidence of the validity of flow theory (ie, that flow is enabled by appropriate challenge). This also implies that studies that neglect to measure or manipulate appropriate challenge may be missing an important antecedent to flow. TVG does not result in flow unless challenge is balanced with skill. Thus, employees who dominate, or get dominated, in the TVG arena may not feel appropriately challenged, which would reduce the likelihood of attaining a state of flow. Future users of TVG for team building should be aware of this.

### Limitations

A few limitations of this research are worth noting. First, this was a laboratory experiment. Although laboratory experiments are a necessary and useful first step in establishing a phenomenon, future research is needed to ensure the results of this study are generalizable to practical workplace settings. One limitation arising from the experimental setting is the artificial time pressure to which we attributed mixed effects of some aspects of flow on team performance.

Managers should not assume all TVG will be beneficial. Each team had a facilitator that encouraged them to participate fully, and the participants may have been motivated by knowing that their cooperation would help the researchers. Therefore, the positive effects of TVG may not be replicated in actual settings if the teams do not engage in the video games.

Our geocaching and tower-building tasks were designed to be enjoyable. The geocaching task was somewhat game-like in that it included a leaderboard and real-time feedback about competitive performance. This may have contributed to the carryover effects of challenge and flow from the TVG treatment to the subsequent tasks. However, we included it to keep our results comparable to prior research on TVG [26]. In contrast, the tower-building task was selected to avoid this bias. There was no leaderboard or competitive feedback, making it more distinct from the TVG treatment. Nevertheless, neither of the tasks were particularly representative of common business work tasks. Therefore, future research should replicate our findings using more generalizable business-oriented tasks.

Another limitation concerns our use of 2 video games. While the video games used represented very different genres, these games have feature sets that represent only some video game characteristics that could be useful as team-building interventions. Future research could map the characteristics of other game features to the traditional team building treatments (interpersonal relations, goal setting, role clarification, problem solving) to see which game types are most effective for each team-building treatment.

Our results were found specifically with participants who were previously unfamiliar with each other, yet interdependent in terms of accomplishing a team task. The TVG treatment may not have the same effect on preexisting teams who have already established norms, biases, and opinions about other team members. In these settings, competitive video gaming may reinforce existing negative biases in relationships that already exist.

Additionally, our participants were college-age students who were generally familiar with video games. The advantage of using student subjects is that (1) it allowed us to replicate the context of new teams and (2) students are typically younger and may be more interested in video gaming than older employees [82]. So, students do not represent all types of employees, and some employees may have negative attitudes toward TVG. Moreover, these students had experience with the games studied in this research. Future research could explore how TVG might work with those who are not familiar with video games. Similarly, if a workplace is not characterized by time-critical, objectively measured tasks, then TVG may become more of a distraction than a team-building activity. Future research should examine how flow works in settings without time pressure.

Finally, although our operationalization of the control group treatment represented a practical example of companies that do not facilitate any sort of team-building activity, it also allowed the opportunity for many possible explanations for the difference with the TVG treatment. For example, participants in the TVG treatment not only got to play video games together but also had the opportunity to develop communication skills and get rewards, whereas the control treatment participants were not allowed to interact in any way. Therefore, we cannot definitively conclude that it was the TVG treatment that caused the performance improvements and not simply the communication or candy rewards. Future research should include additional control treatments that allow basic socializing and communication in between tasks as well as break apart the elements of (1) video gaming, (2) cooperation, and (3) competition to understand the degree to which each element contributes to flow and positive performance effects of TVG.

### Conclusion

Overall, our research contributes to work on TVG outcomes in 3 ways. First, it confirms prior research [26] that found TVG has a positive overall effect on subsequent team performance. Second, flow theory explains significantly more variance in team performance than prior research. TVG creates a norm of finding an appropriate level of challenge and engagement with team tasks. Finally, it demonstrates that flow is a distinct and complementary construct to the traditional team cohesion theory used to explain team performance. We hope our results will inspire additional research into challenge, flow, and the potential benefits of TVG for small work teams.

## Appendix

Multimedia Appendix 1 Measurement model tables and summaries.

Multimedia Appendix 2 Aggregation of individual-level responses into team-level measures.

Multimedia Appendix 3 Differences in mean scores by video game, task, gender, and team size.

Multimedia Appendix 4 CONSORT-eHEALTH checklist (V 1.6.1).

## Abbreviations

ATG-S: Individual Attractions to the Group-Social
ATG-T: individual attractions to the group-task
AVE: average variance extracted
CH: appropriate challenge
CO: control
CU: curiosity
FI: focused immersion
GI-S: Group Integration-Social
GI-T: group integration-task
HE: heightened enjoyment
IS: information system
IT: information technology
PLS: partial least squares
TD: time dissociation
TVG: team video gaming
