# Supplementary material for: Team Building Through Team Video Games: Randomized Controlled Trial
Source: JMIR Serious Games. 2021 Dec 14;9(4):e28896. doi: 10.2196/28896 (PMC8715357; doi:10.2196/28896)
Supplement: Multimedia Appendix 1 [file games_v9i4e28896_app1.docx]

# Appendix 1: Measurement Model Tables and Summaries

| Table A1. Measurement Items and Outer Loadings | | |
| --- | --- | --- |
| *Construct* | *Question* | *Outer loading* |
| Challenge | CH1. Working on the task in my team challenged me to perform to the best of my ability. | 0.770 |
|  | CH2. Working on the task was a good test of my skills. | 0.949 |
|  | CH3. Compared to other team tasks, working on the task stretched my capabilities to my limits. | 0.759 |
| Time distortion | TD1. Time appears to go by very quickly when working on the task in my team. | 0.824 |
|  | TD2. Sometimes I lose track of time when I am working on the task in my team | 0.828 |
|  | TD3. Time flies when I am working on the task in my team | 0.916 |
| Focused immersion | FI1. When working on the task in my team, I am able to block out most other distractions | 0.741 |
|  | FI2. When working on the task in my team, I am absorbed in what I am doing | 0.883 |
|  | FI3. When working on the task in my team, I am immersed in the task I am performing | 0.848 |
|  | FI4. When working on the task in my team, my attention does not get diverted very easily | 0.724 |
| Heightened enjoyment | HE1. I have fun working on the task with my team | 0.911 |
|  | HE2. Working on the task with my team provides me with a lot of enjoyment | 0.922 |
|  | HE3. I enjoy working on the task in my team | 0.904 |
|  | HE4r. Working on the task with my team bores me | dropped |
| Control | CO1. When working on the task in my team, I feel in control | 0.969 |
|  | CO2r. I feel that I have no control when working on the task with my team | dropped |
|  | CO3. I can control my own performance when I am working on the task in my team | 0.651 |
| Curiosity | CU1. Working on the task with my team excites my curiosity | 0.895 |
|  | CU2. Working on the task with my team makes me curious | 0.853 |
|  | CU3. Working on the task in my team arouses my imagination | 0.961 |
| Group interaction | GI-T1r. I am not happy with my level of participation within these activities and what my responsibilities are during the activities | 0.734 |
|  | GI-T2r. I am unhappy with my team’s level of desire to do well in this activity | 0.764 |
|  | GI-T3r. This team does not give me enough opportunities to improve my skills used in these activities | 0.706 |
|  | GI-T4r. I do not like the style of how this team completes this activity | 0.742 |
| Attraction to the group | ATG-T1. Our team is united in trying to reach its goals and performance in this activity | 0.692 |
|  | ATG-T2. We all take responsibility for any loss or poor performance by our team | 0.906 |
|  | ATG-T3r. Our team members have conflicting aspirations for the team’s performance | dropped |
|  | ATG-T4. If members of our team have problems while they are doing this activity everyone wants to help them figure out how to improve their performance | 0.583 |
|  | ATG-T5r. Members of our team do not communicate freely about each other’s performance and abilities during this activity | dropped |
| Interdependence | INT1r. My portion of the task could be performed fairly independently of others. | 0.798 |
|  | INT2r. My portion of the task could be planned with little need to coordinate with others. | 0.768 |
|  | INT3r. My portion of the task was relatively unaffected by the performance of other individuals in my group. | 0.784 |

| Table A2. Reliability, Validity, and Interrater Reliability Criterion for First Order Sub-dimensions | | | | | | | | | | | |
| --- | --- | --- | --- | --- | --- | --- | --- | --- | --- | --- | --- |
|  | ATGT | CH | CO | CU | FI | GIT | HE | INT | TD | Composite  reliability | α |
| ATGT | **0.540** | 0.239 | 0.102 | 0.276 | 0.225 | 0.346 | 0.344 | 0.211 | 0.160 | 0.771 | 0.631 |
| CH | 0.057 | **0.735** | 0.446 | 0.658 | 0.522 | 0.335 | 0.596 | 0.054 | 0.438 | 0.892 | 0.832 |
| CO | 0.010 | 0.199 | **0.722** | 0.480 | 0.488 | 0.322 | 0.471 | -0.102 | 0.259 | 0.838 | 0.628 |
| CU | 0.076 | 0.433 | 0.230 | **0.845** | 0.488 | 0.295 | 0.656 | 0.049 | 0.379 | 0.942 | 0.930 |
| FI | 0.051 | 0.272 | 0.238 | 0.238 | **0.567** | 0.453 | 0.616 | 0.074 | 0.529 | 0.834 | 0.738 |
| GIT | 0.120 | 0.112 | 0.104 | 0.087 | 0.205 | **0.556** | 0.442 | 0.221 | 0.325 | 0.832 | 0.739 |
| HE | 0.118 | 0.355 | 0.222 | 0.430 | 0.379 | 0.195 | **0.852** | 0.072 | 0.446 | 0.945 | 0.916 |
| INT | 0.045 | 0.003 | 0.010 | 0.002 | 0.005 | 0.049 | 0.005 | **0.643** | 0.066 | 0.844 | 0.719 |
| TD | 0.026 | 0.192 | 0.067 | 0.144 | 0.280 | 0.106 | 0.199 | 0.004 | **0.647** | 0.843 | 0.829 |
| **Note:** AVEs are on the diagonal. Correlations are above the diagonal. Squared correlations below the diagonal. ATGT: individual attractions to the group-task, CH: challenge, CO: control, CU: curiosity, FI: focused immersion, GIT: group interaction, HE: heightened enjoyment, INT: Interdependence, TD: temporal dissociation. | | | | | | | | | | | |

| Table A3. Cross-Loading Matrix | | | | | | | | | | | | | | | | | |
| --- | --- | --- | --- | --- | --- | --- | --- | --- | --- | --- | --- | --- | --- | --- | --- | --- | --- |
|  | ATGT | CH | | CO | | CU | | FI | | GI-T | HE | | INT | | TD | | |
| ATG-T1 | **0.779** | 0.212 | | 0.019 | | 0.188 | | 0.193 | | 0.285 | 0.273 | | 0.158 | | 0.139 | | |
| ATG-T2 | **0.868** | 0.175 | | 0.136 | | 0.25 | | 0.179 | | 0.285 | 0.287 | | 0.200 | | 0.127 | | |
| ATG-T4 | **0.509** | 0.197 | | 0.051 | | 0.216 | | 0.13 | | 0.196 | 0.226 | | 0.029 | | 0.087 | | |
| CH1 | 0.162 | **0.821** | | 0.450 | | 0.603 | | 0.563 | | 0.359 | 0.563 | | 0.032 | | 0.413 | | |
| CH2 | 0.254 | **0.951** | | 0.411 | | 0.614 | | 0.441 | | 0.299 | 0.541 | | 0.065 | | 0.391 | | |
| CH3 | 0.162 | **0.791** | | 0.275 | | 0.466 | | 0.379 | | 0.204 | 0.44 | | 0.024 | | 0.345 | | |
| CO1 | 0.153 | 0.392 | | **0.913** | | 0.425 | | 0.417 | | 0.268 | 0.424 | | -0.102 | | 0.214 | | |
| CO3 | -0.013 | 0.371 | | **0.782** | | 0.395 | | 0.424 | | 0.29 | 0.377 | | -0.066 | | 0.236 | | |
| CU1 | 0.250 | 0.621 | | 0.466 | | **0.894** | | 0.479 | | 0.291 | 0.667 | | 0.024 | | 0.363 | | |
| CU2 | 0.242 | 0.643 | | 0.474 | | **0.884** | | 0.469 | | 0.275 | 0.631 | | 0.011 | | 0.323 | | |
| CU3 | 0.269 | 0.619 | | 0.446 | | **0.977** | | 0.453 | | 0.275 | 0.600 | | 0.061 | | 0.363 | | |
| FI1 | 0.129 | 0.477 | | 0.418 | | 0.425 | | **0.774** | | 0.368 | 0.542 | | 0.014 | | 0.455 | | |
| FI2 | 0.163 | 0.508 | | 0.483 | | 0.481 | | **0.919** | | 0.458 | 0.600 | | 0.078 | | 0.530 | | |
| FI3 | 0.273 | 0.226 | | 0.105 | | 0.142 | | **0.496** | | 0.206 | 0.216 | | 0.039 | | 0.171 | | |
| FI5 | 0.125 | 0.359 | | 0.411 | | 0.384 | | **0.760** | | 0.291 | 0.468 | | 0.051 | | 0.392 | | |
| GI-T1 | 0.203 | 0.211 | | 0.267 | | 0.199 | | 0.318 | | **0.647** | 0.320 | | 0.108 | | 0.183 | | |
| GI-T2 | 0.232 | 0.230 | | 0.242 | | 0.210 | | 0.371 | | **0.823** | 0.342 | | 0.213 | | 0.270 | | |
| GI-T3 | 0.264 | 0.246 | | 0.256 | | 0.177 | | 0.320 | | **0.693** | 0.256 | | 0.129 | | 0.234 | | |
| GI-T4 | 0.335 | 0.317 | | 0.228 | | 0.292 | | 0.349 | | **0.806** | 0.397 | | 0.181 | | 0.270 | | |
| HE1 | 0.298 | 0.537 | | 0.446 | | 0.574 | | 0.574 | | 0.434 | **0.919** | | 0.052 | | 0.384 | | |
| HE2 | 0.333 | 0.572 | | 0.419 | | 0.641 | | 0.579 | | 0.402 | **0.947** | | 0.085 | | 0.434 | | |
| HE3 | 0.316 | 0.535 | | 0.456 | | 0.589 | | 0.555 | | 0.398 | **0.902** | | 0.052 | | 0.41 | | |
| INT1 | 0.191 | 0.027 | | -0.154 | | 0.001 | | -0.029 | | 0.131 | 0.023 | | **0.830** | | 0.032 | | |
| INT2 | 0.132 | 0.027 | | -0.074 | | 0.029 | | 0.082 | | 0.165 | 0.018 | | **0.782** | | 0.06 | | |
| INT3 | 0.18 | 0.075 | | -0.015 | | 0.087 | | 0.13 | | 0.235 | 0.128 | | **0.793** | | 0.067 | | |
| TD1 | 0.111 | 0.395 | | 0.317 | | 0.345 | | 0.553 | | 0.322 | 0.462 | | -0.009 | | **0.625** | | |
| TD2 | 0.125 | 0.366 | | 0.163 | | 0.308 | | 0.418 | | 0.251 | 0.338 | | 0.062 | | **0.941** | | |
| TD3 | 0.172 | 0.454 | | 0.366 | | 0.406 | | 0.599 | | 0.374 | 0.527 | | 0.042 | | **0.816** | | |
| Table A4. Reliability and Validity Criterion for Highest Level Constructs | | | | | | | | | | | | | | | |  |  |
|  | | | TF | | TC | | CH | | INT | Composite  reliability | | α | | VIF | | |  |
| Team flow (TF) | | | **0.937** | | 0.760 | | 0.907 | | 0.863 | 0.987 | | 0.983 | | 1.301 | | |  |
| Team cohesion (TC) | | | 0.578 | | **0.918** | | 0.780 | | 0.867 | 0.978 | | 0.970 | | 1.235 | | |  |
| Challenge (CH) | | | 0.823 | | 0.608 | | **0.789** | | 0.873 | 0.917 | | 0.873 | | 1.173 | | |  |
| Interdependence (INT) | | | 0.745 | | 0.751 | | 0.772 | | **0.726** | 0.837 | | 0.752 | | n/a | | |  |
| **Note:** AVEs are on the diagonal. Correlations are above the diagonal. Squared correlations are below the diagonal. | | | | | | | | | | | | | | | |  |  |
