# Supplementary material for: Team Building Through Team Video Games: Randomized Controlled Trial
Source: JMIR Serious Games. 2021 Dec 14;9(4):e28896. doi: 10.2196/28896 (PMC8715357; doi:10.2196/28896)
Supplement: Multimedia Appendix 2 [file games_v9i4e28896_app2.docx]

**Appendix 2: Aggregation of Individual-level Responses into Team-level Measures**

High interrater agreement (IRA) justifies aggregation. Judges should generally agree on judgments on a variable [1, 2]. This reflects good construct-level validity [1]. We used r_WG(J)_, which indicates the extent to which team members’ responses to survey questions converge greater than would be expected by chance James, Demaree [3]. As part of this measure, observed variance across multiple respondents is compared to the maximum expected variance that would result from completely random ratings, $\sigma_{EU}^{2}$, which is 4.0 for a uniform distribution. Because our data reflected a slight optimistic rating bias, we also show results compared to $\sigma_{EU}^{2}$= 2.9, a slightly skewed distribution [4]. LeBreton and Senter [4] suggested that r_WG(J)_ levels of .71-.90 are strong agreement and levels of .91 to 1.00 are very strong agreement. Interrater reliability values for this study are shown in Table A5. We found very strong or strong levels of agreement when compared to both expected random distributions.

| Table A5. Within-Group Interrater Reliabilities | | | | | | | | | | |
| --- | --- | --- | --- | --- | --- | --- | --- | --- | --- | --- |
| Null | Mean | SD | Mean | SD | Mean | SD | Mean | SD | Mean | SD |
| Distribution | ATGT | | CH | | CO | | CU | | FI | |
| r_WG(j).uniform_ | .93 | .05 | .95 | .04 | .93 | .06 | .94 | .04 | .95 | .07 |
| r_WG(j).sskewed_ | .88 | .09 | .87 | .08 | .88 | .10 | .89 | .07 | .92 | .16 |
|  | GIT | | HE | | INT | | TD | |  |  |
| r_WG(j).uniform_ | .95 | .03 | .95 | .04 | .90 | .04 | .95 | .04 |  |  |
| r_WG(j).sskewed_ | .89 | .04 | .92 | .07 | .84 | .07 | .91 | .07 |  |  |

Also to justify aggregation, group membership should matter to scores within the group and across groups and there should be good reliability of measurement within and across groups [5, 6]. The intraclass correlation coefficients ICC(1) and ICC(2) indices “are the two most frequently adopted reliability measures for the single score and average score ICCs, respectively, within the context of multilevel modeling” [7, p. 594]. ICCs were calculated using formulae from prior research [1, 8]. ICC(1) and ICC(2) scores reflect the level of observed variance of a single score of an individual within a team and the variance of the average scores that result from aggregation of the individual scores, respectively. Woehr, Loignon [6] reviewed ICC(1) and ICC(2) scores in 416 published articles that measured team-level constructs in multilevel studies. The average ICC(1) value in published studies was 0.21 (SD=.15). The average ICC(2) level was 0.66 (SD=.18). Table A6 shows the values calculated in the current study.

| Table A6. Intraclass Correlation Coefficients | | | | | | | | | |
| --- | --- | --- | --- | --- | --- | --- | --- | --- | --- |
| ICC | ATGT | CH | CO | CU | FI | GIT | HE | INT | TD |
| ICC(1) | .16 | .65 | .09 | .24 | .07 | .25 | .16 | .70 | .25 |
| ICC(2) | .43 | .88 | .28 | .55 | .24 | .57 | .43 | .90 | .57 |
| F-Stat | 1.753 | 8.283 | 1.381 | 2.235 | 1.321 | 2.322 | 1.752 | 10.517 | 1.609 |
| Significance | *P*<.001 | *P*<.001 | *P*<.01 | *P*<.001 | *P*<.05 | *P*<.001 | *P*<.001 | *P*<.001 | *P*<.001 |
| **Note**: ATGT: individual attractions to the group-task, CH: challenge, CO: control, CU: curiosity, FI: focused immersion, GIT: group interaction, HE: heightened enjoyment, INT: interdependence, TD: temporal dissociation. | | | | | | | | | |

All measures of ICC(1) and ICC(2) were statistically significant. The ICC(1) measures on seven (ATGT, CH, CU, FI, FIT, HE, INT, TD) of nine constructs were above or near the mean of published studies. The ICC(1) values for the remaining two constructs (CO and FI) were within one standard deviation below the mean. ICC(2) values are a function of group size. The average group size in the present study was four which results in a lower ICC(2) value than for studies based on larger groups. The ICC(2) measures on two (CH and INT) of nine constructs were greater than one standard deviation above the mean of published studies. Three constructs (CU, GIT, and TD) were less than one standard deviation below the mean. Two (ATGT and HE) constructs were slightly below one standard deviation below the mean, and two (CO and FI) were more than two standard deviations below the mean. Considering these indices, we concluded aggregation was justified for these variables.

1. James LR. Aggregation bias in estimates of perceptual agreement. Journal of applied psychology. 1982;67(2):219.

2. Klein DN, Ouimette PC, Kelly HS, Ferro T, Riso LP. Test-retest reliability of team consensus best-estimate diagonses of Axis I and II disorders in a family study. The American Journal of Psychiatry. 1994.

3. James LR, Demaree RG, Wolf GJJoap. Estimating within-group interrater reliability with and without response bias. 1984;69(1):85.

4. LeBreton JM, Senter JL. Answers to 20 questions about interrater reliability and interrater agreement. Organizational research methods. 2008;11(4):815-52.

5. Van Mierlo H, Vermunt JK, Rutte CGJORM. Composing group-level constructs from individual-level survey data. 2009;12(2):368-92.

6. Woehr DJ, Loignon AC, Schmidt PB, Loughry ML, Ohland MW. Justifying aggregation with consensus-based constructs: A review and examination of cutoff values for common aggregation indices. Organizational Research Methods. 2015;18(4):704-37.

7. Shieh G. Choosing the best index for the average score intraclass correlation coefficient. Behavior research methods. 2016;48(3):994-1003.

8. Bliese PD. Within-group agreement, non-independence, and reliability: Implications for data aggregation and analysis. 2000.
