# Supplementary material for: Team Building Through Team Video Games: Randomized Controlled Trial
Source: JMIR Serious Games. 2021 Dec 14;9(4):e28896. doi: 10.2196/28896 (PMC8715357; doi:10.2196/28896)
Supplement: Multimedia Appendix 3 [file games_v9i4e28896_app3.docx]

**Appendix 3. Differences in Mean Scores by Video Game, Task, Gender, and Team Size**

We examined differences in means scores by video game, task, gender, and team size. Each p-value is based on an ANOVA controlling for all other variables (see Table A7). *P*-values <= .10 are bolded.

| Table A7. Summary of Descriptive Means and Differences | | | | | | | | | | | | | | | | | |
| --- | --- | --- | --- | --- | --- | --- | --- | --- | --- | --- | --- | --- | --- | --- | --- | --- | --- |
|  | **Video Game** | | | | | | **Gender** | | | | | | **Team Size** | | | | |
|  | R. Band | | Halo | | *p* | | Female | | Male | | *p* | | 3 | | 4 | | *p* |
| % task Δ | 44.0% | 68.5% | | **<.001** | | 61.2% | | 48.2% | | **<.05** | | 60.7% | | 49.6% | | **<.01** | |
| % female | 29.9% | 18.8% | | **<.001** | | 100% | | 0% | | n/a | | 24.3% | | 22.7% | | .68 | |
| Pre-Test Scores | | | | | | | | | | | | | | | | | |
| Challenge | 4.72 | 4.88 | | .687 | | 4.58 | | 4.83 | | **<.05** | | 4.54 | | 4.81 | | **.080** | |
| Flow: CO | 5.11 | 5.22 | | .887 | | 5.09 | | 5.16 | | .278 | | 5.10 | | 5.15 | | .685 | |
| Flow: CU | 5.08 | 5.36 | | **.066** | | 5.10 | | 5.18 | | .384 | | 4.99 | | 5.20 | | .359 | |
| Flow: FI | 5.63 | 5.69 | | .356 | | 5.71 | | 5.63 | | .967 | | 5.61 | | 5.65 | | .841 | |
| Flow: HE | 5.50 | 5.60 | | .886 | | 5.60 | | 5.51 | | .183 | | 5.47 | | 5.54 | | .462 | |
| Flow: TD | 5.78 | 6.04 | | **<.001** | | 5.88 | | 5.85 | | .842 | | 5.85 | | 5.86 | | .260 | |
| Interdependence | 3.97 | 4.01 | | .990 | | 4.27 | | 3.95 | | **<.001** | | 3.96 | | 4.04 | | .607 | |
| Team cohesion | 4.61 | 4.54 | | .144 | | 4.65 | | 4.56 | | .152 | | 4.59 | | 4.58 | | .824 | |
| *Post-Test Scores* | | | | | | | | | | | | | | | | | |
| Challenge | 4.63 | 4.77 | | .751 | | 4.62 | | 4.68 | | .584 | | 4.57 | | 4.69 | | .990 | |
| Flow: CO | 5.40 | 5.53 | | .516 | | 5.48 | | 5.42 | | .465 | | 5.39 | | 5.44 | | .904 | |
| Flow: CU | 5.13 | 5.33 | | .219 | | 5.08 | | 5.16 | | .388 | | 5.00 | | 5.17 | | .946 | |
| Flow: FI | 5.82 | 5.91 | | .146 | | 5.82 | | 5.91 | | .439 | | 5.83 | | 5.85 | | .410 | |
| Flow: HE | 5.69 | 5.82 | | .814 | | 5.88 | | 5.68 | | **<.05** | | 5.63 | | 5.75 | | .745 | |
| Flow: TD | 5.80 | 5.99 | | .821 | | 5.97 | | 5.83 | | .798 | | 5.93 | | 5.85 | | .103 | |
| Interdependence | 3.86 | 3.86 | | .723 | | 3.91 | | 3.85 | | **.071** | | 3.91 | | 3.85 | | .832 | |
| Team cohesion | 5.49 | 5.39 | | **<.05** | | 5.69 | | 5.39 | | **<.001** | | 5.53 | | 5.45 | | .555 | |

As shown in the table’s first row, those who played Halo demonstrated a somewhat greater increase in performance from the pretest to posttest than those who play Rock Band (*P* =.001). In addition, females were more likely to choose to play Rock Band than Halo (*P*=.001). Halo players exhibited moderately higher curiosity (*P*=.066) and much greater time dissociation (*P*<.001) during the pre-test, but lower team cohesion after the post-test (*P*<.05).

The two tasks differed greatly, which provides insights into how the team video gaming treatment related to task. Task 1, geocaching, took longer to complete, required participants to identify and physically go to landmarks, and was more complicated than Task 2 (tower building). Task 1 produced greater levels of all components of flow and greater team cohesion before and after the team video gaming treatment. No significant difference was found for interdependence between the tasks on the pretest, but Task 1 led to greater interdependence during the posttest.

There were several gender differences. First, females improved performance more than males. Upon closer examination, females started with lower Task 1 performance scores, but finished with very similar scores to males in Task 2—thus explaining the larger change in percent improvement. This may suggest that 1) our tasks were more suited to the skills of the males in our samples, but also, 2) that females benefited greater from the team video gaming intervention. Females tended to work in a more interdependent manner than males. In addition, females expressed more heightened enjoyment from the tasks than males.

While most of the teams included four members, there were occasional “no shows” by individuals scheduled to participate in the study: thus, resulting in about 20% of the teams including only 3 members. However, there were almost no differences between 3- and 4-person teams. However, 4-person teams perceived greater challenge and curiosity during the pretest task. There were no differences from the posttest task.
